# Association between pre-stroke frailty status and post-stroke cognitive impairment in patients with acute large artery atherosclerotic cerebral infarction

**DOI:** 10.1590/1414-431X2025e14837

**Published:** 2025-09-12

**Authors:** Jun Wang, Yanrong Yuan, Yan Zhang, Huili Liu, Yongxing Yan

**Affiliations:** 1 Department of Neurology, Hangzhou Third People's Hospital, Hangzhou, Zhejiang, China

**Keywords:** Cerebral infarction, TOAST, Cognitive impairment, Frailty

## Abstract

The aim of this study was to investigate the correlation between pre-stroke frailty status and post-stroke cognitive impairment (PSCI) in patients with acute large artery atherosclerotic cerebral infarction. One hundred and eight patients with acute large artery atherosclerotic cerebral infarction admitted in our hospital from July 2020 to July 2023 were prospectively enrolled. Patients were stratified into frailty (46 cases) and non-frailty groups (62 cases) based on FRAIL scale scores. During the 6-month follow-up after the onset of cerebral infarction, patients were evaluated using the Chinese modified version of Montreal Cognitive Assessment (MoCA) scale for cognitive function and were divided into PSCI (52 cases) and non-PSCI (56 cases) groups. The frailty group showed significantly higher age, prevalence of hypertension and diabetes comorbidities, smoking and alcohol consumption rates, National Institutes of Health Stroke Scale (NHISS) score, and Modified Rankin Scale (mRS) score than those in the non-frailty group (P<0.05, P<0.01). The incidence of PSCI in the frailty group was also significantly higher than that in the non-frailty group (78.3 *vs* 25.8%, P<0.01). Compared to the non-PSCI group, the PSCI group had higher age, shorter education duration, fewer cases of reperfusion therapy, and greater frailty (P<0.05, P<0.01). Logistic regression analysis showed that pre-stroke frailty was an independent risk factor for PSCI (P<0.01). Timely assessment of the frailty status in patients with acute large artery atherosclerotic cerebral infarction is beneficial for preventing, delaying onset, and reducing the incidence of PSCI.

## Introduction

The high incidence, mortality, and disability rates of ischemic stroke represent a major public health burden. Post-stroke cognitive impairment (PSCI), a common complication of ischemic stroke, refers to a clinical syndrome characterized by cognitive impairment that occurs within 6 months after the stroke event (1). Approximately 7.4% of stroke patients experience immediate cognitive impairment, whereas 30 to 88.1% of patients develop PSCI (2-4). In addition to cognitive and social dysfunction, PSCI can impede rehabilitation of physical or language functions, further reducing the quality of life of patients and increasing the burden on families, caregivers, and the society (5). However, unlike Alzheimer disease (AD) - a progressive neurodegenerative disorder - PSCI is triggered by stroke events and often exhibits a fluctuating disease course, with some patients even experiencing reversible cognitive impairment. More importantly, ischemic stroke-induced PSCI is both preventable and treatable. Early identification and timely intervention can not only delay and even reverse cognitive decline, but also improve the patients' daily living abilities and reduce mortality. Currently, most PSCI patients at diagnosis have missed the golden window of early intervention, underscoring the critical need for clinicians to recognize PSCI at its earliest stages.

Frailty is a prevalent geriatric syndrome, a clinical state with diminished physiological reserve across multiple systems, leading to increased vulnerability and decreased stress resistance in older adults (6,7). The introduction of the concept of frailty and its clinical application have assisted disease evaluation and prognosis assessment in the elderly, as well as provided novel therapeutic approaches and management strategies. Current research shows that frailty is associated with cardiovascular disease, diabetes, chronic kidney disease, and other elderly chronic diseases and to the prognosis of these diseases (8-10). Studies have also shown that frailty is associated with decreased cognitive function in the elderly (11,12). Given PSCI's significance as a key prevention and control target for ischemic stroke, the potential correlation between frailty and PSCI warrants further investigation.

According to the TOAST (Trial of ORG 10172 in Acute Stroke Treatment) criteria, all acute cerebral infarction patients can be divided into five subtypes. The etiology of cerebral infarction in different subtypes varies, with the large artery atherosclerotic (LAA) etiology being the most common cause. Therefore, this study aimed to preliminarily explore the correlation between pre-stroke frailty status and PSCI in patients with acute LAA cerebral infarction in order to provide a theoretical basis for early recognition and intervention of PSCI.

## Material and Methods

### Subjects

Patients who experienced an initial episode of acute LAA cerebral infarction admitted to the Department of Neurology of Hangzhou Third People's Hospital from July 2020 to July 2023 were prospectively enrolled. All patients met the diagnostic criteria for acute ischemic stroke as outlined in the 2018 Chinese Guidelines for the Diagnosis and Treatment of Acute Ischemic Stroke (13).

Inclusion criteria were: 1) age≥60 years; 2) having clear clinical symptoms and signs of stroke; 3) first-time onset of ischemic stroke; 4) within 48 h of onset; 5) LAA cerebral infarction diagnosis according to TOAST classification.

Exclusion criteria were: 1) transient ischemic attack; 2) individuals with aphasia, visual/auditory impairments, consciousness disorders, etc., who were unable to cooperate in completing frailty and cognitive function assessments; 3) patients with cognitive impairment before hospitalization; 4) individuals with severe anxiety, depression, and mental disorders in the past; 5) individuals with severe heart, lung, liver, and kidney dysfunction.

This study was approved by the Ethics Committee of Hangzhou Third People's Hospital (approval code: 2020KA015), and all study subjects signed informed consent forms.

### Clinical data collection

A database of relevant clinical data was established for enrolled patients. General information of patients was collected, including age, gender, duration of education, and disease comorbidities such as hypertension, diabetes, coronary heart disease, atrial fibrillation, chronic obstructive pulmonary disease (COPD), etc. Additionally, data on smoking habits (defined as an average of ≥1 cigarette/day for a continuous period of ≥6 months), alcohol consumption (defined as an average intake of ≥100 g of alcohol/day, for a duration of ≥1 year) were gathered. Clinical assessments including the National Institutes of Health Stroke Scale (NIHSS) score and the Modified Rankin Scale (mRS) score were conducted during hospitalization by qualified neurologists.

### Frailty assessment

Patients underwent a frailty status assessment within 24 h of admission by qualified neurologists using the FRAIL scale, as proposed by the International Association for Nutrition and Aging (14). This scale comprises five criteria: fatigue, resistance, ambulation, illness, and weight loss. Each criterion is assessed through a specific question: “Have you often felt fatigued in the past month?”, “Did you have difficulty climbing a flight of stairs without rest or assistance prior to this stroke?”, “Did you have difficulty walking a block without rest or assistance prior to this stroke?”, “Do you have more than five of the following conditions: hypertension, diabetes, cancer, COPD, myocardial infarction, congestive heart failure, angina, asthma, arthritis, stroke, and kidney disease?”, and “Have you lost more than 5% of your body weight in the past year?”. To each “yes” response to a question is assigned 1 point, while a “no” response is assigned 0 points. A total score of 3 or more points indicates frailty.

### Cognitive function assessment

Within 24 h after admission and 6 months after stroke onset, cognitive function was evaluated using the Chinese version of Montreal Cognitive Assessment (MoCA) scale (15) by qualified neurologists. The scale includes 11 test items in 8 cognitive domains, including attention and concentration, executive function, memory, language, visual structural skills, abstract thinking, computation, and directional ability. The scale total score is 30 points. If the subject's education level is less than 12 years, 1 point is added to the final result. If the score is less than 26 points, the subject is considered to have cognitive impairment.

### Statistical analysis

SPSS 16.0 software (IBM, USA) was used for statistical processing of the data. The Kolmogorov-Smirnov test was used to distinguish normal from abnormal distribuions of the quantitative data. The normally distributed quantitative variables are reported as means±SD, and an independent sample *t*-test was used for inter-group comparison. Categorical data are reported as frequency and percentage (%), and comparisons between groups are performed using χ^2^ test or Fisher's exact test. Univariate analysis was applied to screen the associated factors in PSCI and non-PSCI patients, while multiple logistic regression analysis was applied to identify independent risk factors for PSCI. The impact of clinical factors (duration of education, comorbidities, etc.) was also assessed. In all tests, P<0.05 was considered statistically significant.

## Results

### General clinical data of frailty and non-frailty groups

**Figure 1:**
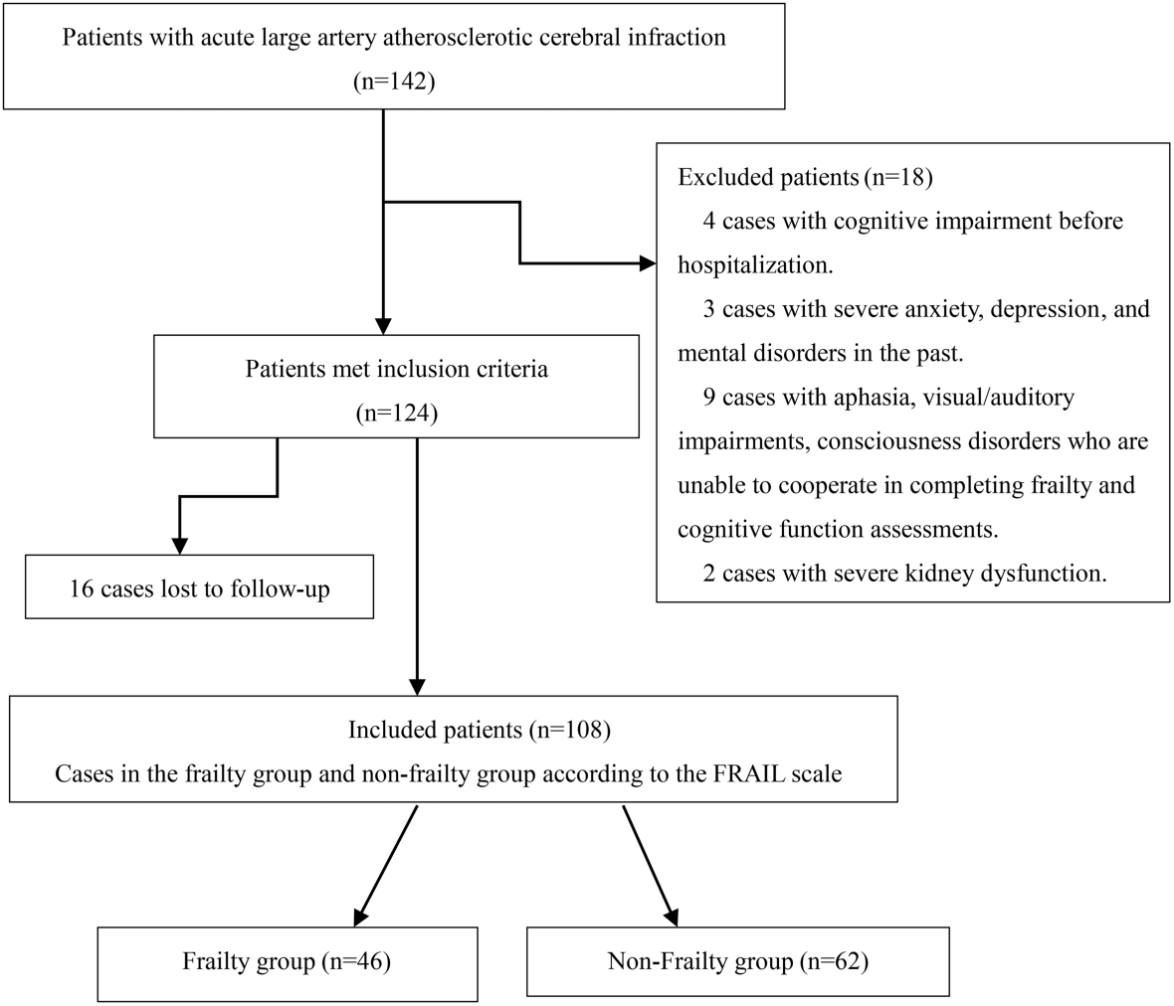
Study flowchart.

In 142 cases of acute LAA cerebral infarction, 18 patients were excluded based on the criteria. Of the 124 patients initially enrolled, 16 were lost to follow-up, and 108 patients were ultimately selected, including 80 males and 28 females, with an average age of 72.5±7.6 years (60-90 years) (Figure 1).

Of the 108 patients with acute LAA cerebral infarction, 46 were frailty patients according to FRAIL score at admission and 62 were non-frailty. No statistically significant differences were observed in gender, duration of education, and comorbidities (coronary heart disease, atrial fibrillation, COPD and reperfusion therapy) between the two groups (P>0.05). However, patients in the frailty group exhibited significantly higher age, prevalence of hypertension, diabetes, smoking, alcohol consumption, and NHISS and mRS scores at admission than those in the non-frailty group (P<0.05, P<0.01; Table 1).

**Table 1:** Comparison of baseline characteristics between frailty and non-frailty groups.

	Frailty group(n=46)	Non-frailty group (n=62)	*t*/*χ* ^2^	P
Age (years)	75.1±8.0	70.6±6.7	3.190	0.002
Gender (n)				
Male	32	48	0.848	0.357
Female	14	14		
Duration of education (years)	7.1±3.0	7.4±4.0	0.380	0.705
Comorbidity (n)				
Hypertension	37	25	4.601	0.032
Diabetes	17	29	5.401	0.020
Coronary heart disease	12	13	0.241	0.623
Atrial fibrillation	2	5	0.532	0.466
COPD	8	16	0.696	0.404
Smoking (n)	25	14	5.309	0.021
Alcohol consumption (n)	19	11	4.054	0.044
NIHSS score at admission	9.4±4.4	7.5±2.5	2.923	0.004
mRS score at admission	2.8±1.3	2.3±1.1	2.401	0.018
Reperfusion therapy (n)	7	7	0.3610	0.5480
IVT	5	4	0.6747	0.4114
IVT+EMT	7	7	0.0144	0.5480

Data are reported as mean±SD or number (n). COPD: chronic obstructive pulmonary disease; mRS: Modified Rankin Scale; NIHSS: National Institutes of Health Stroke Scale; IVT: Intravenous thrombolysis; EMT: endovascular mechanical thrombectomy. The independent sample *t*-test, χ^2^ test, or Fisher's exact statistical test were used to determine significance.

### Cognitive function of frailty and non-frailty patients

The MoCA score of patients in the frailty group at admission was slightly lower than that of the non-frailty group (27.1±1.3 *vs* 27.5±1.4), but the difference was not statistically significant (P>0.05). After 6 months of follow-up, the MoCA total score of the frailty group was significantly lower than that of the non-frailty group (23.0±2.8 *vs* 25.5±2.5) (P<0.01). Within the frailty group, 36 patients had PSCI (36/46, 78.3%), whereas in the non-frailty group, 16 patients had PSCI (16/62, 25.8%) (P<0.01). Both groups showed a notable decline in MoCA total score after 6 months of follow-up compared to their cognitive function at admission (P<0.01), mainly due to decline of visual spatial executive function, naming ability, attention, delayed recall, and directional ability (P<0.05, P<0.01), as summarized in Table 2.

**Table 2:** Comparison of cognitive function scores between frailty and non-frailty groups.

	Frailty group (n=46)		Non-frailty group (n=62)	
	At admission	6 months follow-up	At admission	6 months follow-up
Chinese version of MoCA total score	27.1±1.3	23.0±2.8^##^	27.5±1.4	25.5±2.5**^##^
Visual spatial execution function	3.7±0.9	2.8±1.3^##^	4.1±0.9*	3.5±1.0**^##^
Naming	2.9±0.3	2.7±0.5^#^	2.7±0.4	2.7±0.5
Attention	5.1±0.7	4.2±0.9^##^	5.2±0.6	4.8±1.0**^#^
Language	2.8±0.5	2.6±0.6	2.7±0.5	2.7±0.5
Abstract ability	1.9±0.3	1.9±0.3	1.9±0.2	1.9±0.3
Delayed recall	4.5±0.6	3.6±0.6^##^	4.5±0.6	4.0±0.7**^##^
Directional ability	5.6±0.5	4.6±0.8^##^	5.8±0.4	5.4±0.7**^##^

Data are reported as mean±SD. *P<0.05. **P<0.01 compared with the frailty group in the same period; ^#^P<0.05, ^##^P<0.01 compared with at admission. MoCA: Montreal Cognitive Assessment scale. The independent sample Student's *t*-test was used to determine significance.

### General clinical data of PSCI group and non-PSCI group patients

According to MoCA scale scores at 6 months follow-up, out of 108 patients with acute LAA cerebral infarction, 52 (48.1%) developed PSCI (PSCI group), while 56 cases (51.9%) did not develop PSCI (non-PSCI group). Compared with the non-PSCI group, the PSCI group had significantly higher age, lower duration of education, more severe frailty, and higher rates of hypertension and diabetes (P<0.05). However, there was no significant difference in gender, other comorbidities such as coronary heart disease, atrial fibrillation, smoking, alcohol consumption, and NIHSS and mRS scores (P>0.05). Moreover, there were more cases receiving reperfusion therapy in the non-PSCI group than in the PSCI group (P<0.05), while there was no significant difference in the number of cases receiving intravenous thrombolysis (IVT) and endovascular mechanical thrombectomy (EMT) alone (P>0.05) Table 3.

**Table 3:** Comparison of clinical characteristics between post-stroke cognitive impairment (PSCI) group and non-PSCI group.

	PSCI group(n=52)	Non-PSCI group (n=56)	*t*/*χ* ^2^	P
Age (years)	77.2±6.7	68.2±5.5	7.634	0.001
Gender (n)				
Male	34 (65.4%)	44 (78.6%)	2.337	0.126
Female	18 (34.6%)	12 (21.4%)		
Years of education	5.8±3.5	8.6±3.2	4.283	0.001
Comorbidity (n)				
Hypertension	40 (76.9%)	22 (39.3%)	4.250	0.039
Diabetes	32 (61.5%)	14 (25.0%)	5.968	0.015
Coronary heart disease	10 (19.2%)	15 (26.8%)	0.542	0.462
Atrial fibrillation	4 (7.7%)	3 (5.4%)	0.213	0.645
COPD	13 (25.0%)	11 (19.6%)	0.285	0.594
Smoking (n)	23 (44.2%)	16 (28.6%)	1.344	0.246
Alcohol consumption (n)	18 (34.6%)	12 (21.4%)	1.319	0.251
NIHSS score at admission	9.1±4.3	7.8±2.4	1.900	0.060
mRS score at admission	2.8±1.2	2.6±1.0	1.046	0.298
Frailty (n)	36 (69.2%)	10 (17.9%)	11.945	0.001
Reperfusion therapy (n)	3 (5.7%)	11 (19.6%)	4.600	0.032
IVT	2 (3.8%)	7 (12.5%)	2.643	0.104
IVT+EMT	1 (1.9%)	4 (7.1%)	1.664	0.197
Chinese version of MoCA total score				
At admission	26.9±1.0	27.3±1.5	1.618	0.109
6 months follow-up	21.8±1.8	26.9±0.8	19.630	0.001

Data are reported as means±SD or number (%). COPD: chronic obstructive pulmonary disease; IVT: intravenous thrombolysis; EMT: endovascular mechanical thrombectomy. mRS: Modified Rankin Scale; IVT: NIHSS: National Institutes of Health Stroke Scale; MoCA: Montreal Cognitive Assessment scale; IVT: Intravenous thrombolysis; EMT: endovascular mechanical thrombectomy. The independent sample Student's *t*-test, χ^2^ test, or Fisher's exact tests were used to determine significance.

### Logistic regression analysis

Parameters with significant differences in the intergroup comparison (age, duration of education, comorbidities including hypertension and diabetes, frailty, reperfusion therapy) were used as the covariates in the logistic regression. After adjusting for duration of education and comorbidities, the results showed that age, comorbidity with diabetes, and frailty were independent risk factors for PSCI occurrence. Conversely, reperfusion therapy was identified as a protective factor, as summarized in Table 4.

**Table 4:** Multivariate logistic regression analysis of post-stroke cognitive impairment (PSCI) occurrence.

Variable	β value	SE value	Wald	OR value	95%CI	P value
Age	0.182	0.071	6.595	1.199	1.044-1.378	0.010
Duration of education	-0.104	0.125	0.687	0.902	0.706-1.152	0.407
Comorbidity hypertension	1.148	0.683	2.826	3.152	0.827-12.019	0.093
Comorbidity diabetes	1.978	0.736	7.216	7.225	1.707-30.586	0.007
Frailty	2.857	0.743	14.768	17.411	4.055-74.758	0.000
Reperfusion therapy	-2.023	0.945	4.585	0.132	0.021-0.843	0.032

## Discussion

Frailty is a clinical condition hallmarked by progressive decline of multiple physiological systems, leading to a compromised physiological reserve, impaired homeostasis maintenance, increased vulnerability, and a decreased stress resistance (6,16). Frailty can also increase the risk of adverse consequences, including falls, delirium, disability, hospitalization, and even death (6,17,18). Therefore, frailty can serve as a predictive indicator for adverse disease outcomes in elderly individuals, including disability, cognitive decline, and death (17,19). However, frailty can be prevented, delayed, or even reversed by administering medication and/or non-medication interventions. Therefore, early identification, timely evaluation of the patient's frailty status, and active intervention are of great significance.

At present, the frailty status of patients is mainly evaluated through scales, such as the Fried phenotype assessment, frailty index, FRAIL scale, and clinical frailty scale. Notably, the FRAIL scale, proposed by the International Association for Nutrition and Aging, does not require complex examination items. Compared with other evaluation methods, it is both quick and simple, allowing for self-reported responses. Consequently, the FRAIL scale was preferred by healthcare professionals for early identification of frail patients and to guide interventions. Studies demonstrated that the FRAIL scale performs comparably to comprehensive assessment tools, like the Fried phenotype, in evaluating and predicting adverse health outcomes among older adults (20,21). Given that this study involved patients with acute ischemic stroke who were unable to complete complex physical function tests, we utilized the FRAIL scale for frailty measurement. We measured frailty within 24 h of admission to determine pre-stroke frailty status. Of 108 patients with acute LAA cerebral infarction, 46 patients were frail before the stroke, accounting for 42.6% cases, which was higher than the 17.4% frailty rate in the elderly population of 1235 in Chinese communities reported by Dong et al. (22). We postulated that the higher frailty state was due to the fact that the subjects in this study were all over 60 years of age and were acute ischemic stroke patients with multiple system diseases and cerebrovascular risk factors. In this study, age, NIHSS, and mRS scores at admission of the frailty group were significantly higher than those in the non-frailty group, indicating that acute LAA cerebral infarction patients with frailty had more severe neurological deficits and disabilities compared to non-frailty patients. In agreement with previous studies, the age of frail patients was greater than that of non-frail individuals (23).

An increasing number of studies have shown a close relationship between frailty and cognitive impairment in recent years. A Canadian study identified frailty as an important risk factor for dementia in patients with mild cognitive impairment (MCI) (24). A systematic review by Vahedi et al. (25) found a strong correlation between frailty and cognitive dysfunction. Siejka et al. (26) also found that frailty was an important factor for early cognitive impairment, and suggested that measuring frailty can help to identify the risk of future cognitive decline. Consistent with previous findings, this study found that the proportion of cognitive impairment in the pre-stroke frailty group was significantly higher than that in non-frailty group after 6 months of follow-up, further confirming the close association between frailty and cognitive impairment, although the stroke patients included in this study did not exhibit cognitive impairment in the early stages.

PSCI is one of the common complications of stroke, and the cognitive function of PSCI patients may further deteriorate, thereby seriously affecting the quality of life and survival. At present, there is no clinically specific treatment for PSCI. However, if patients at high risk for PSCI can be identified through early and accurate assessment followed by preventive interventions, PSCI can be prevented. The cognitive deficits of PSCI mainly manifest as impairments in executive function, memory, and orientation.

The MoCA was designed by Nasreddine et al. (27) to screen elderly MCI patients. It encompasses a comprehensive range of cognitive domains and effectively reflects the neuropsychological impairments of patients. It has high sensitivity and specificity to identify cognitive impairment and has been widely used worldwide. This scale was translated into Chinese by Wei et al. (28,29) in 2007 with the consent of the authors and has been widely used for screening cognitive function in the Chinese population. Using this Chinese version of the MoCA scale in this study, we also found that regardless of frailty status, patients have a cognitive decline after 6 months of the ischemic stroke with impaired visual spatial executive function, naming ability, attention, delayed recall, and orientation. Taylor-Rowan et al. (30) found that pre-stroke frailty is independently associated with PSCI in a cross-sectional study.

In our study, the PSCI group exhibited higher age, lower duration of education, higher frailty status, and more comorbidities with hypertension and diabetes than the non-PSCI group. Further logistic regression analysis revealed that, in addition to age, duration of education and comorbidities with diabetes were independent risk factors for PSCI, which was recognized by other studies (31-33). The pre-stroke frailty status also emerged as an independent risk factor for PSCI, which further explained the relationship between frailty and PSCI. Patients with pre-stroke frailty were more susceptible to develop PSCI, potentially because of advanced age, comorbidities with more underlying diseases, and frailty-related cognitive impairment in the central nervous system, warranting further research. Therefore, clinicians need to pay attention to the relationship between the frailty status of patients with acute LAA cerebral infarction and PSCI, which has certain value for the early prediction of PSCI.

IVT and EMT are important reperfusion treatment methods for acute ischemic stroke (AIS) patients and have been shown to be very effective in improving patient prognosis. Theoretically, reperfusion therapy promotes vascular recanalization, restores central nervous system blood supply, and may contribute to the improvement of cognitive function. However, there are inconsistent reports regarding the efficacy of reperfusion therapy on cognitive function in patients with AIS. Some studies have found that both IVT and EMT can substantially improve cognitive function in AIS patients (34,35). Conversely, a retrospective study by Torrisi et al. (36) involving 92 AIS patients found no significant difference in the incidence of cognitive impairment between the IVT group and the non-IVT group at 3 months post-onset. Additionally, another retrospective analysis showed that thrombolytic therapy improved acute cognitive function in AIS, but did not improve long-term cognitive function (37). This study also found that reperfusion therapy is an independent protective factor for the occurrence of PSCI. However, the limited number of patients receiving reperfusion therapy in our study precluded the detection of statistical differences between the PSCI and non-PSCI groups when IVT or EMT alone was used. Consequently, further exploration is needed to elucidate the impact of reperfusion therapy on PSCI.

The present study had certain limitations. First, it was a single-center study with a small sample size, which may introduce a selection bias. Secondly, this study did not use alternative frailty assessment methods for comparative analysis, nor did it investigate the impact of frailty on stroke location, size, and other related factors. In addition, cognitive function includes multiple cognitive domains, and different scales have different focuses on evaluating cognitive function. For example, the Trail Making Test (TMT) A and TMT B focus on assessing patients' attention, reaction speed, and executive function, while the Boston Naming Test (BNT) evaluates language function in stroke patients. This study only used one scale to assess the overall cognitive function of patients, which may have overlooked patients with individual decline in cognitive domains not covered by the instrument. Future research needs to address this limitation by assessing individual cognitive domains. Moreover, multi-center and large-sample clinical studies are needed to further clarify our results and provide reference for early targeted intervention in PSCI.

## Conclusion

PSCI has a high incidence rate and insidious onset, representing a prevalent neurovascular dysfunction after stroke and seriously affects the living standards of patients. The pre-stroke frailty status of patients with acute LAA cerebral infarction can predict an increased risk of PSCI, which suggests that the frailty status of ischemic stroke patients can be timely evaluated with suitable assessment tools such as the FRAIL scale after stroke. Such evaluations can serve as early warning and monitoring mechanisms for PSCI occurrence, thereby helping clinicians in developing more comprehensive strategies to intervene and delay the occurrence of PSCI.
